# Detection of surface defect on flexible printed circuit via guided box improvement in GA-Faster-RCNN network

**DOI:** 10.1371/journal.pone.0295400

**Published:** 2023-12-05

**Authors:** Xiaole Shen, Yunlong Xing, Jinhui Lu, Fei Yu

**Affiliations:** 1 College of Big Data and Internet, Shenzhen Technology University, Shenzhen, Guang Dong, China; 2 School of Electronics and Communication Engineering, Shenzhen Polytechnic University, Shenzhen, Guang Dong, China; 3 School of Integrated Circuits, Shenzhen Polytechnic University, Shenzhen, Guang Dong, China; VIT-AP Campus, INDIA

## Abstract

Industrial defect detection is a critical aspect of production. Traditional industrial inspection algorithms often face challenges with low detection accuracy. In recent years, the adoption of deep learning algorithms, particularly Convolutional Neural Networks (CNNs), has shown remarkable success in the field of computer vision. Our research primarily focused on developing a defect detection algorithm for the surface of Flexible Printed Circuit (FPC) boards. To address the challenges of detecting small objects and objects with extreme aspect ratios in FPC defect detection for surface, we proposed a guided box improvement approach based on the GA-Faster-RCNN network. This approach involves refining bounding box predictions to enhance the precision and efficiency of defect detection in Faster-RCNN network. Through experiments, we verified that our designed GA-Faster-RCNN network achieved an impressive accuracy rate of 91.1%, representing an 8.5% improvement in detection accuracy compared to the baseline model.

## Introduction

With the development of new-generation electronic technologies such as lithography alignment techniques based on Moiré Fringe [1], 3D printed for silver mesh [2], and the advancement of mathematical principles in electronic engineering [3], there has been a significant focus on the fabrication of Flexible Transparent Electrodes (FTE), which is a critical component in electronic devices [4, 5]. Another closely related component, FPC, has also garnered extensive attention due to its lightweight, thin profile, and the ability to bend and fold freely.

**Fig 1:**
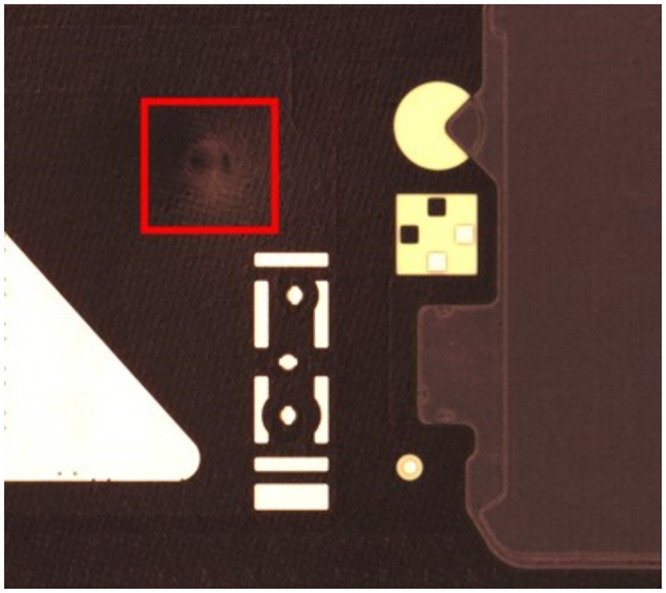
Depression example.

**Fig 2:**
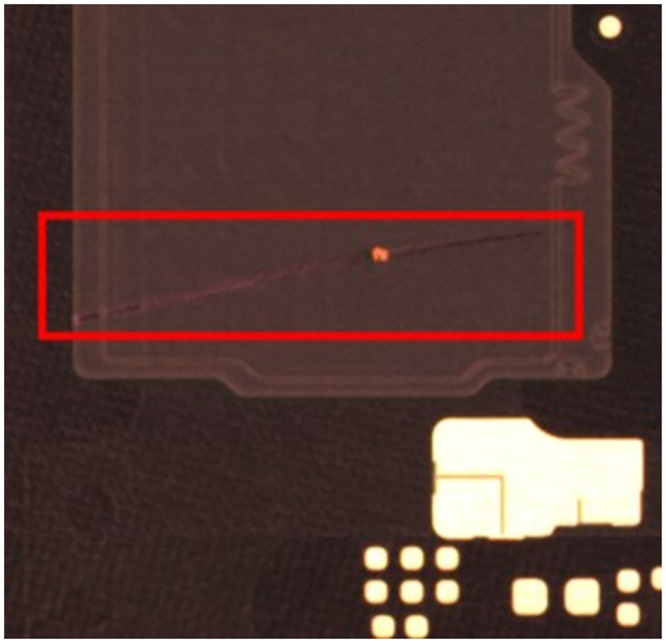
Protrusion example.

In the production of FPC, various defects can arise due to process-related issues. These defects can be categorized into six types, including depressions, protrusions, contamination, discoloration, incomplete penetration, and cutting misalignment, as depicted in Figs 1 and 2. Enhancing the accuracy of circuit board defect detection is of paramount importance in ensuring the quality of products, making it a central focus in the field of FPC defect inspection today.

In the early stages, defects in PCBs were detected through manual visual inspection. However, this method gradually gave way to Automatic Optical Inspection (AOI) technology. As PCB surface textures become increasingly complex, research on defect detection for PCB surface has been ongoing. However, since FPCs emerged later than regular PCBs, most of the current research is based on regular PCBs, and the algorithms defect detection for surface of FPCs largely rely on those used for regular PCBs. These algorithms primarily consist of two categories: classical image processing algorithms and neural network algorithms.

### Research on classical image processing algorithms

Image processing algorithms are at the core of machine vision algorithms. To address the problem of defect detection for PCB, many researchers have proposed their solutions. Li YF and Li SY, considering the limitations of using a single feature to describe various defects on bare PCB, proposed a defect detection algorithm based on gradient direction information entropy and uniform local binary patterns [6]. In addition, Lu ZS et al., pointed out that most detection algorithms rely on comparison methods, which are not effective in extreme cases, and proposed a non-reference comparison framework for defect detection for PCB [7]. Tsai DM and Huang CK, focused on defect detection in non-periodic patterns and designed a global Fourier image reconstruction method [8]. Cai L and Li JC emphasized image preprocessing algorithms and detection analysis [9]. They proposed an image preprocessing and detection algorithm to address the limitations of existing traditional detection methods and demonstrated its good detection performance and high accuracy.

In general, machine vision approaches using various image processing algorithms and techniques are employed for detecting and locating PCB defects. However, these methods also have limitations, such as low detection accuracy. As a result, many researchers have begun to explore the use of deep learning methods to address this issue.

### Research on neural network algorithms

Currently, in the field of defect detection for surface, machine learning and deep learning algorithms based on neural networks are more popular compared to classical image processing algorithms. Zhang C et al., proposed an algorithm for defect detection for surface of PCB based on deep feature learning [10], which reduces the demand for large-scale datasets while improving detection accuracy. Ran GZ et al., recognized the low robustness issue in defect detection algorithms and proposed a defect detection algorithm for PCB based on the Single Shot Multibox Detector (SSD) framework of convolutional neural networks [11]. Hu B and Wang JH, combined the needs of practical PCB production and proposed an improved Faster-RCNN network model [12] based on Faster-RCNN [13]. It uses a ResNet-50 network [14] with feature pyramids as the backbone for feature extraction, utilizes GARPN to predict more accurate anchors, and merges residual units of ShuffleNetV2, achieving good detection performance. Zhang H et al., presented the Cost-Sensitive Residual Convolutional Neural Network (CS-ResNet) model [15], which effectively addresses the issue of imbalanced class distribution and misclassification of true and false defects. Adibhatla VA et al., proposed a novel unsupervised learning method that utilizes teacher-student feature pyramid matching as a pre-trained image classification model to learn the distribution of normal images and detect defects in PCB, effectively addressing the problem of requiring a large amount of manual annotation data in models like YOLO [16]. Wan YS et al., addressed the issue of small sample labeling in defect detection for PCB and proposed a semi-supervised defect detection method (DE-SSD) based on a data expansion strategy. It achieves good accuracy in defect detection for PCB with fewer labeled samples [17].

In conclusion, deep learning algorithms have been widely applied in the field of defect detection for surface, Especially Faster-RCNN network, which uses ResNet-50 network with feature pyramids as the backbone for feature extraction, predicts more accurate anchors with the help of GARPN, and merges residual units of ShuffleNetV2. However, there are still significant gaps in detecting small targets and extreme aspect ratio defects in defect detection for surface of FPC. To address this gap, we have designed an improved GA-Faster-RCNN network based on guided bounding boxes to tackle this issue.

## Materials and methods

**Fig 3:**
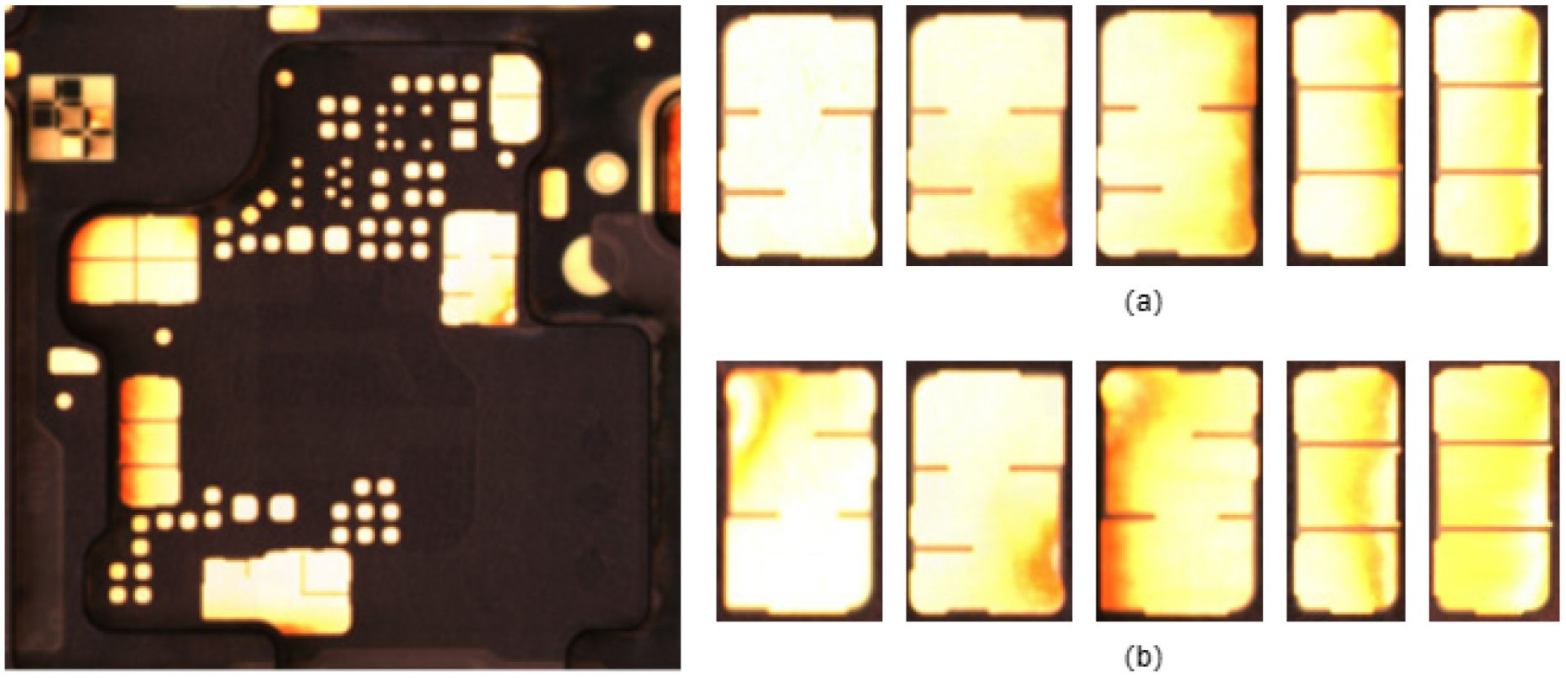
Defect examples.

In FPC surface defects, some types of defects occur randomly in terms of location. For example, defects such as bubbles, inclusions, and indentations that arise from production processes can exhibit random positioning. These defects can be classified into two categories: protrusion defects and depression defects. We created a defect dataset based on existing data, which consists of 3,992 images of protrusion defects and 5,025 images of depression defects. Fig 3 presents examples of these two types of defects, where a, b, and c represent protrusion defects, and d, e, and f represent depression defects.

We propose a GA-Faster-RCNN network model for FPC surface defects with small targets, multiple sizes, and extreme aspect ratios. We use the Guided Anchoring (GA) [18] module to replace the RPN network in the original Faster-RCNN, and increase the feature extraction capability of multi-scale defects with the help of the Feature Pyramid Network(FPN) [19] network, and replace the VGG16 [20] in the original Faster-RCNN with ResNet-101 network. The GA-Faster-RCNN network model can solve the above problems well with the help of the FPN network to increase the ability of multi-scale defect extraction, and the VGG16 in the original Faster-RCNN is replaced by the ResNet-101 network, which is experimentally verified to have an accuracy improvement of 8.5 percentage points compared with the Faster-RCNN.

### GA-Faster-RCNN network architecture

**Fig 4:**
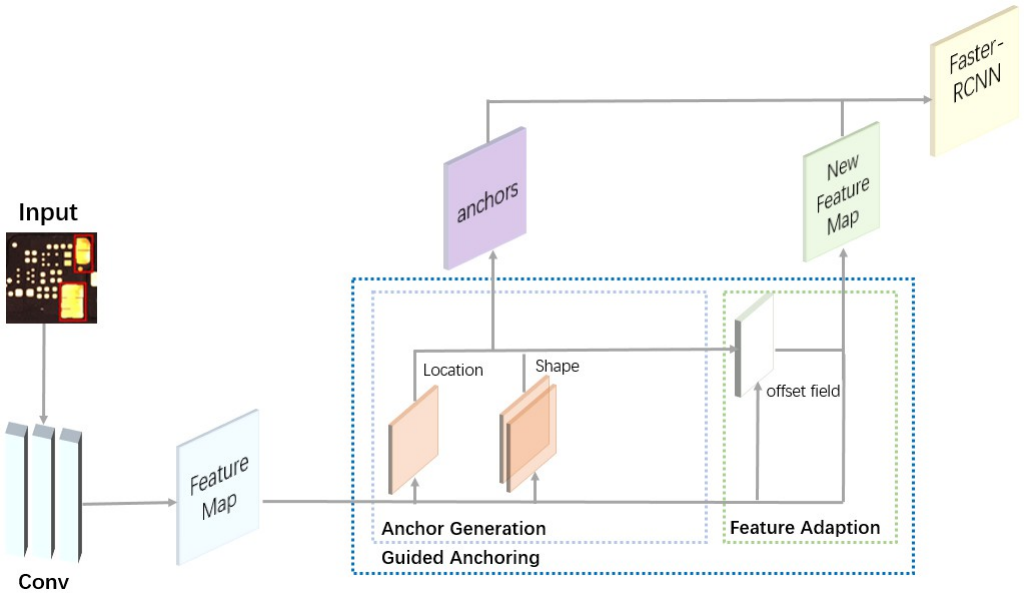
GA-Faster-RCNN network architecture.

The GA-Faster-RCNN network framework is illustrated in Fig 4. It begins with feature extraction using convolutional layers, where we used the ResNet-101 network known for its powerful feature extraction capabilities. Within the convolutional layers, we introduced the FPN to enhance multi-scale feature extraction. The extracted feature maps from ResNet-101 were then fed into the position prediction branch and shape prediction branch. The position prediction branch predicts which regions should serve as anchor centers, while the shape prediction branch uses Intersection over Union (IoU) to guide the network in learning the optimal length and width given the anchor centers. This approach allows the learned anchors to adapt to extreme cases without being limited by predefined values. The generated anchors, along with the new feature maps, were subsequently fed into Faster-RCNN for classification predictions. Through experimental verification, our improved network exhibits superior accuracy in detecting multi-scale defects, achieving an accuracy rate of 91.1%.

### GA algorithm

The GA module allows for the direct training of sparse and shape-variable anchors. In general, anchors can be represented by four parameters (x, y, w, h), which correspond to the coordinates of the anchor’s center point as well as its width and height. The probability distribution of anchors can be decomposed into two conditional probability distributions, described in Eq 1.

$$\begin{array}{c} p(x,y,w,h|I)=p(x,y|I)p(w,h|x,y,I) \end{array}$$ (1)

**Fig 5:**
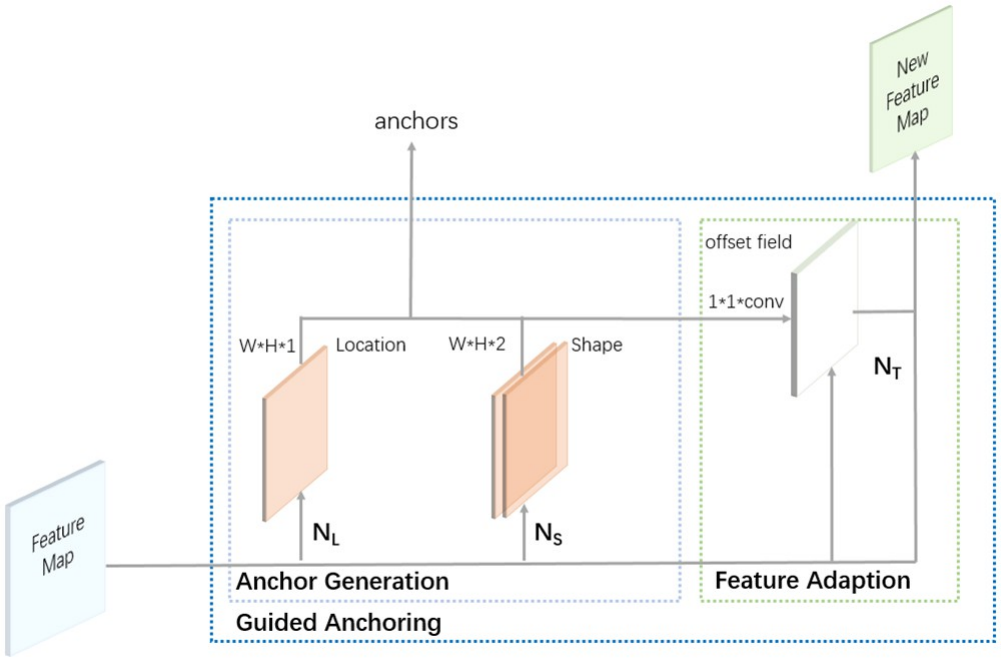
Guided anchoring structure.

*p*(*x*, *y*, *w*, *h*|*I*) represents the probability distribution of anchor center points given the feature map, and *p*(*w*, *h*|*x*, *y*, *I*) represents the probability distribution of anchor width and height given the feature map and center point. According to Eq 1, the GA module is designed with two sub-networks: the location prediction sub-network *N*_*L*_ and the shape prediction sub-network *N*_*S*_. The structure of the GA module is illustrated in Fig 5.

#### Location prediction

The objective of the location prediction branch in GA is to predict which regions should be selected as anchor center points. In the location prediction sub-network, the entire feature map is divided into object center regions. peripheral regions, and ignored regions. The region corresponding to the center of the ground truth box on the feature map is marked as the object center region and treated as a positive sample during training. The remaining regions are labeled as ignored or negative samples based on their distance from the center. Through position prediction, a small subset of regions can be selected as candidate anchor center points, significantly reducing the number of anchors.

#### Shape prediction

The purpose of the shape prediction branch is to predict the optimal length and width of an anchor given its center point as the size of defects are intricate. IoU is used as the supervision to learn the values of width and height. Since IoU is differentiable, the network can be trained to maximize the IoU value. The matching between anchors and ground truth is represented by the following Eq 2:

$$\begin{array}{c} vIoU\left(a_{wh},gt\right)=max_{w>0,h>0}IoU_{normal}\left(a_{wh},gt\right) \end{array}$$ (2)

*a*_*wh*_ represents the width and height of the anchor, and *gt* represents the ground truth value. Because of the large range of *w* and *h* when learning them directly, they are transformed using the following Eq 3:

$$\begin{array}{c} w=\sigma \cdot s\cdot e^{dw},h=\sigma \cdot s\cdot e^{dh} \end{array}$$ (3)

*σ* is an empirical scale factor (set to 8), and *s* is the stride. The shape prediction branch outputs *dw* and *dh*, which are then mapped to *w* and *h*.

#### Feature adaption

In the RPN, anchors are uniformly distributed across the entire image in a sliding window-like manner, and each anchor is associated with a feature map of the same size. However, in GA, the size of anchors is not fixed, and it is necessary to use features with different receptive fields to leverage the advantages of different anchor sizes. To achieve this, feature adaptation is required, and its formula is Eq 4:

$$\begin{array}{c} f_{i}=N_{T}\left(f_{i},w_{i},h_{i}\right) \end{array}$$ (4)

*N*_*T*_ represents a 3 × 3 deformable convolution, and *i* is the index of each anchor. The shape information generated by the shape prediction sub-network is incorporated into the original feature map through the deformable convolution. Finally, we employ a multi-task loss for end-to-end training, with the loss function is Eq 5:

$$\begin{array}{c} L=\lambda_{1}L_{loc}+\lambda_{2}L_{shape}+L_{cls}+L_{reg} \end{array}$$ (5)

*L*_*cls*_ represents the classification loss, *L*_*reg*_ represents the regression loss, *L*_*loc*_ represents the location prediction loss, and *L*_*shape*_ represents the shape prediction loss.

### FPN

In Faster-RCNN, the RPN is typically applied to the last feature map for candidate box extraction. However, the last feature map, after multiple downsampling operations, may lose some features of small objects. Therefore, even with the integration of the GA module in Faster-RCNN, the missed small objects cannot be detected. To address this issue, we introduce the FPN.

**Fig 6:**
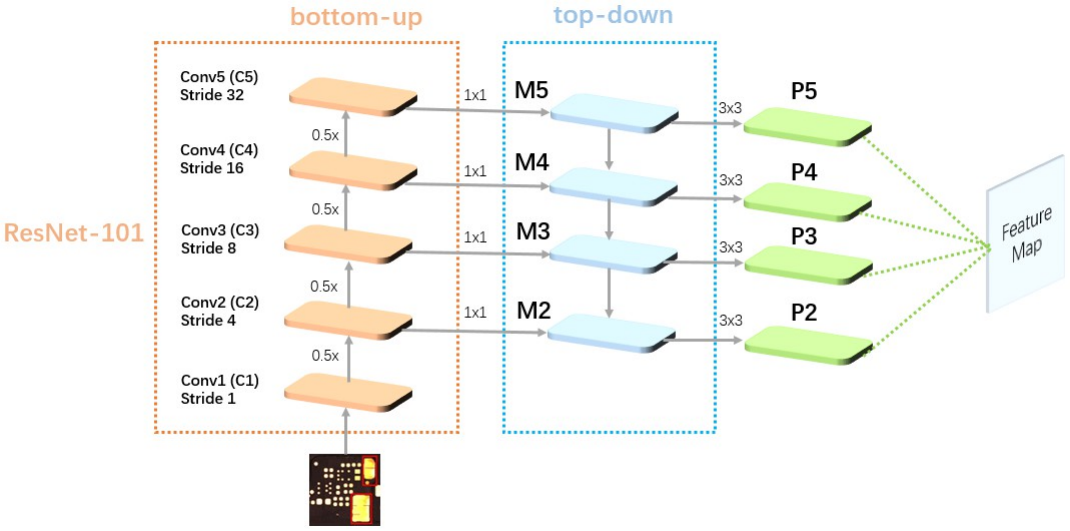
FPN network structure.

The FPN module is a deep neural network designed to handle images of different scales. Its main purpose is to construct a feature pyramid at different scales, combining high-level semantic information (smaller receptive fields) with low-level detailed information (larger receptive fields), to obtain a more comprehensive and rich feature representation. The FPN module consists of two main parts: bottom-up and top-down. We place the FPN module at the end of the ResNet-101 network, using FPN to obtain a feature pyramid with multi-scale feature information. The overall structure is illustrated in Fig 6.

In the bottom-up process, the original image is passed through convolutional layers (Conv2-Conv5) to generate feature maps (C2, C3, C4, C5). Then, the higher-level feature maps with more abstract and semantically rich information are fused with the lower-level feature maps that contain more details about smaller objects. These newly generated feature maps contain more comprehensive information. In the top-down process, the upper-level feature maps are fused with the lower-level feature maps using 1 × 1 convolutions, and then 3 × 3 convolutions are applied to generate P2-P5. Finally, the feature maps obtained from FPN, which contain multi-scale information, are fed into the GA module for more effective detection of smaller defects.

## Experiments

Our proposed GA-Faster-RCNN replaces the RPN in the original Faster-RCNN with the GA module to generate fewer, but more accurate anchors. To address the issue of poor feature extraction for small targets due to the significant variation in defect sizes, we incorporated the FPN network. To evaluate the performance of the model, we conducted two types of experiments. The first type was ablation experiments, which compared the improved Faster-RCNN with its original version by analyzing the contributions of each module. The second type was comparative experiments, which compared GA-Faster-RCNN with other mainstream defect detection networks. Ultimately, our experimental results demonstrate that our designed network achieved an improvement of more than 8% in accuracy.

### Settings

The experiments were conducted on a machine with the following specifications: Ubuntu 20.04.3 LTS operating system, 64GB RAM, NVIDIA Quadro graphics processor, and Intel(R) Xeon(R) Gold 5118 CPU @ 2.30GHz 2.29GHz. The implementation of the proposed network model was based on Python 3.8.10 using PyTorch, OpenCV, and CUDA Toolkit. The hyperparameters of the model were determined through training the network. The settings for network training parameters are shown in Table 1:

**Table 1:** Network training parameters.

Parameters	Value
Learning rate	0.0025
Momentum	0.9
Weight Decay	0.0001
Epochs	24
Etc.	default

### Evaluation metrics

In object detection, commonly used evaluation metrics are precision and recall, but there is a slight difference compared to classification tasks caused by the presence of bounding boxes. In object detection, IoU is used to measure the overlap between two regions. IoU is calculated as the ratio of the intersection area of the ground truth and prediction to the union area of the two regions. Eq 6 describes IoU:

$$\begin{array}{c} IoU=\frac{area\;of\;overlap}{area\;of\;union} \end{array}$$ (6)

In this case, precision and recall in object detection are defined by incorporating IoU. When calculating precision and recall, the following parameters are still used:

TP (True Positive): Predicted bounding boxes that correctly detect the defect class and have an IoU greater than the threshold.

FP (False Positive): Predicted bounding boxes that either incorrectly detect the defect class with an IoU below the threshold or detect the wrong defect class.

FN (False Negative): Predicted bounding boxes that detect the wrong defect class.

TN (True Negative): Predicted bounding boxes that detect a different defect class, which is actually the correct defect class.

The formulas for Precision and Recall are Eqs 7 and 8:

$$\begin{array}{c} Precision=\frac{TP}{TP\;+\;FP} \end{array}$$ (7)

$$\begin{array}{c} Recall=\frac{TP}{TP\;+\;FN} \end{array}$$ (8)

The precision-recall curve, often referred to as the P-R curve, can be plotted with precision on the y-axis and recall on the x-axis. The area enclosed by this curve and the coordinate axes is called the Average Precision (AP). In object detection, AP is commonly used as a comprehensive evaluation metric for models. It is calculated using the following Eq 9:

$$\begin{array}{c} AP=\int_{0}^{1}p\left(r\right)dr \end{array}$$ (9)

AP is an evaluation metric for the detection results of a specific defect class. It is common to use mean Average Precision (mAP) to evaluate the detection performance across multiple defect classes. Eq 10 describes mAP:

$$\begin{array}{c} mAP=\frac{\sum_{i=1}^{n}AP_{i}}{n} \end{array}$$ (10)

*n* is the total number of defect classes, and *i* is the index of the defect class.

## Results

We proposed an improved GA-Faster-RCNN algorithm based on guided anchoring for the detection of depression and protrusion defects in FPC surface. Additionally, we enhanced the feature extraction capability of the network for small-sized defects by incorporating the FPN network. Finally, through quantitative and qualitative analysis using ablation experiments and cross-validation experiments, we validated the effectiveness of our proposed algorithm.

### Ablation experiments

**Fig 7:**
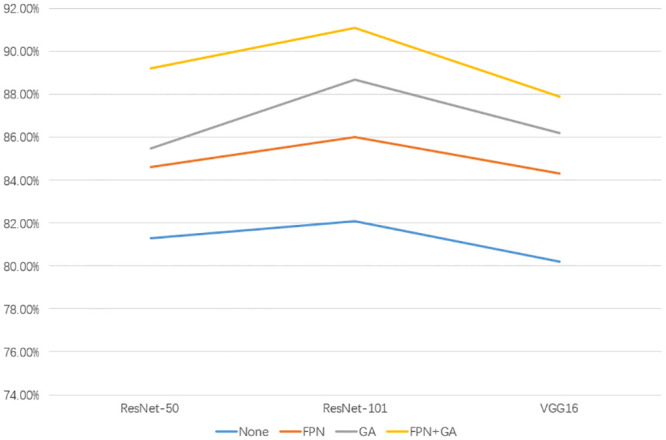
Results for different combinations in ablation experiments.

We conducted ablation experiments to evaluate the improvements made to the original Faster-RCNN. By combining different network models and functional modules, we obtained the experimental results shown in Table 2 and Fig 7.

**Table 2:** Experimental results for different combinations in ablation experiments.

FPN	GA	mAP%		
		ResNet-50	ResNet-101	VGG16
✕	✕	81.3	82.1	80.2
✔	✕	84.6	86.0	84.3
✕	✔	85.5	88.7	86.2
✔	✔	89.2	91.1	87.9

Table 2 indicates that the inclusion of FPN and GA led to a significant improvement in the accuracy of Faster-RCNN. When both modules were incorporated, the improvement ranged from 7% to 9% depending on the feature extraction network. Furthermore, removing either module individually resulted in a decrease in accuracy by at least 1%. When the ResNet-101 was chosen as the feature extraction network, the accuracy improvement ranged from 1% to 3% compared to other feature extraction networks under the same conditions.

### Cross-validation experiments

**Fig 8:**
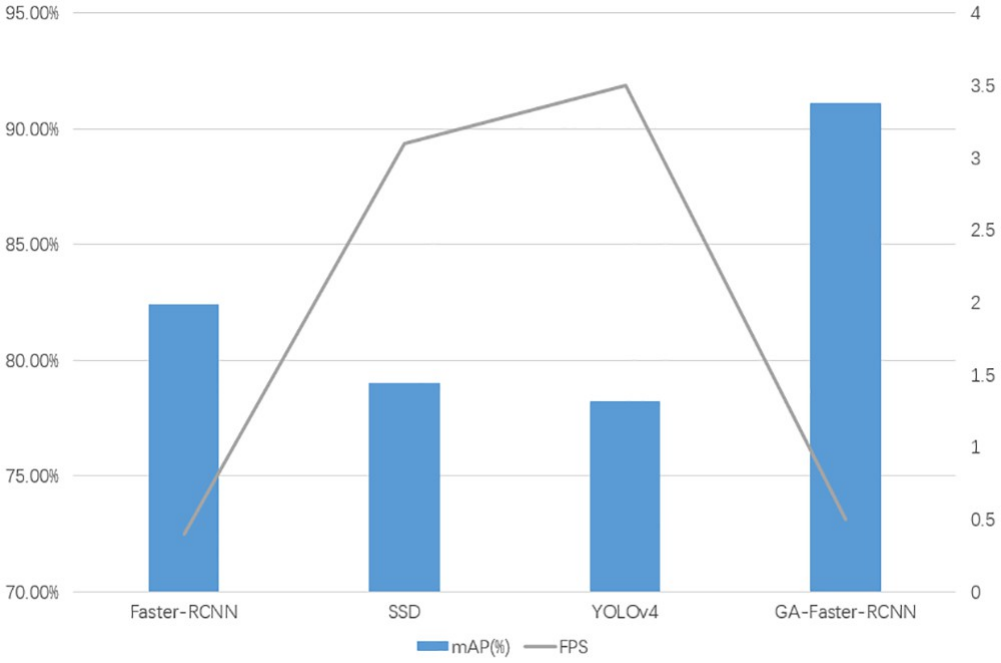
Cross-validation experiments results.

To validate the effectiveness of our proposed model, we conducted comparative experiments with mainstream object detection algorithms currently used in the industry, using different datasets. The experimental results are shown in Table 3 and Fig 8:

**Table 3:** Cross-validation experiments results.

Network	mAP(%)	FPS
Faster-RCNN	82.4	0.4
SSD	79.0	3.1
YOLOv4	78.2	3.5
GA-Faster-RCNN	91.1	0.5

Table 3 shows that our designed GA-Faster-RCNN network achieved a significant improvement in accuracy compared to mainstream object detection algorithms. The average precision reached 91.1%, which is an improvement of 8.5% over the original Faster-RCNN network. The improvement in accuracy was more than 10% compared to typical SSD and YOLOv4 models. However, it should be noted that the GA-Faster-RCNN network had a slight decrease in Frames Per Second (FPS) compared to SSD and YOLOv4. Nonetheless, it still exhibited a slight improvement compared to the original Faster-RCNN network.

## Discussion

The GA-Faster-RCNN network integrates GA, FPN, and ResNet-101 to enhance the ability of the original Faster-RCNN to detect surface defects. It enables the network to predict the location and shape of defects through self-training and uses more comprehensive defect feature representations, which allows previously challenging defects to be recognized by the network. Additionally, in practical industrial production, extreme defects are relatively rare. When using conventional detection algorithms on vast amounts of data, these specific defects may be overlooked because of their lower weights, and prevent the network from learning the detection and recognition capability for these specific defects. However, the self-training approach through the GA module can partially address this issue.

The results validate the effectiveness of the proposed GA-Faster-RCNN network model. It mainly addresses the issues of small-sample training and low accuracy in identifying small targets in defect detection tasks, which may reduce the cost of defect detection. The accuracy exceeding 90% and the improvement of more than 8% demonstrate the successful improvements of the GA-Faster-RCNN network model over the original Faster-RCNN. The conducted ablation experiments confirmed the effectiveness of incorporating the FPN module to address the issue of missing small targets. By fusing more abstract feature maps with stronger semantics and feature maps with more small targets, incorporating the information-rich newly generated feature maps, and using effective anchors generated by the GA module for training assistance, both modules play important roles in improving accuracy.

However, because of the GA-Faster-RCNN network model’s reliance on the GA module for self-training to generate effective anchors, which have variable sizes, the network’s performance in terms of FPS lags behind conventional algorithms. Nonetheless, compared to the baseline Faster-RCNN, there is still a slight improvement. Considering the significant improvement in accuracy and the specific needs of industrial scenarios, where accuracy is often more critical than FPS, the designed GA-Faster-RCNN network model has great significance for FPC industrial defect detection.

Although the experiments have demonstrated a significant improvement in accuracy with the proposed GA-Faster-RCNN network model, the FPS indicates that there is still room for improvement. Additionally, the scarcity of FPC industrial datasets may result in discrepancies between the network’s performance in experiments and its real-world application. Therefore, future improvements can focus on simplifying the overall network architecture and adopting data generation methods that better align with practical industrial environments.

## Conclusion

We conducted research on the integration of defect detection algorithms and deep learning for defect detection for surface in FPC production. Based on the characteristics of defects in FPC products, we conducted in-depth research on defect detection algorithms for surface. By specifically targeting the detection of small objects and objects with extreme aspect ratios, we improved the algorithms. Through ablation experiments, we demonstrated the effectiveness of our designed GA-Faster-RCNN network, with ResNet101 as the feature extraction network, showing excellent capabilities. Furthermore, through cross-comparison experiments, we validated the reliability of the model, achieving an mAP of 91.1%, which is an 8.5% improvement compared to the original Faster-RCNN. This approach can provide effective assistance in both practical and industrial contexts.
